# Coherent optical phonon oscillation and possible electronic softening in WTe_2_ crystals

**DOI:** 10.1038/srep30487

**Published:** 2016-07-26

**Authors:** Bin He, Chunfeng Zhang, Weida Zhu, Yufeng Li, Shenghua Liu, Xiyu Zhu, Xuewei Wu, Xiaoyong Wang, Hai-hu Wen, Min Xiao

**Affiliations:** 1 National Laboratory of Solid State Microstructures, School of Physics, Nanjing University, Nanjing 210093, China; 2 Synergetic Innovation Center in Quantum Information and Quantum Physics, University of Science and Technology of China, Hefei, Anhui 230026, China; 3 Department of Physics, University of Arkansas, Fayetteville, Arkansas 72701, United States

## Abstract

A rapidly-growing interest in WTe_2_ has been triggered by the giant magnetoresistance effect discovered in this unique system. While many efforts have been made towards uncovering the electron- and spin-relevant mechanisms, the role of lattice vibration remains poorly understood. Here, we study the coherent vibrational dynamics in WTe_2_ crystals by using ultrafast pump-probe spectroscopy. The oscillation signal in time domain in WTe_2_ has been ascribed as due to the coherent dynamics of the lowest energy A_1_ optical phonons with polarization- and wavelength-dependent measurements. With increasing temperature, the phonon energy decreases due to anharmonic decay of the optical phonons into acoustic phonons. Moreover, a significant drop (15%) of the phonon energy with increasing pump power is observed which is possibly caused by the lattice anharmonicity induced by electronic excitation and phonon-phonon interaction.

Intensive research interest has been triggered in recent years by the discovery of extremely large magnetoresistance (MR) in tungsten ditelluride (WTe_2_)^1^. The non-saturating MR in this semimetal has been connected to the electronic structure near Fermi surface with equal amount of electrons and holes^1^,^2^ similar to that in semimetals of bismuth^3^ and PtSn_4_^4^. Moreover, intriguing features with strong spin-orbit interaction^5^ and type-II Weyl points^6^ at the Fermi surface have been observed, suggesting the existence of exotic electronic transport properties with promising application potentials for WTe_2_. While many efforts have been made towards understanding the physics relevant to electronic and spin states^2^,^5^,^7^,^8^,^9^,^10^,^11^,^12^,^13^,^14^,^15^, the temperature dependence^16^, three-dimensional anisotropy of MR effect^13^,^16^, and the drastic pressure effect on the MR effect^17^ and superconductivity^18^,^19^ show the important roles played by the lattice dynamics in this system, which, however, has been rarely investigated^20^,^21^,^22^.

Raman scattering spectroscopy is a conventional approach to study lattice dynamics in condensed matter. A recent Raman spectroscopic study on WTe_2_ crystals has reported 7 out of the 33 optical modes (5A_1_ + 2A_2_) predicted by first-principle calculations with *C*_*2v*_ point group symmetry^20^. It has been argued that the symmetric spectra of these phononic modes suggest the absence of strong electron-phonon or spin-phonon coupling^20^. In the past decades, an alternative tool of time-resolved pump-probe spectroscopy has been developed to study the dynamics of non-equilibrium phonons in solids^23^,^24^. The coherent lattice dynamics manifested as oscillations in time-resolved reflected signals is essential for understanding the coherent acoustic and optical phonons, which is particularly suitable for the low-energy regimes being inaccessible for the conventional Raman spectroscopy^25^,^26^,^27^,^28^,^29^. In the well-known nonmagnetic MR material of bismuth, the pump-probe spectroscopy has been widely employed to probe and control the coherent phonons that are susceptible to the small atomic displacement, the lattice defects and bond softening^30^,^31^,^32^,^33^,^34^,^35^,^36^. Time-resolved study can probe the dynamics of anharmonic phonons due to the electronic softening^37^,^38^,^39^ with anomaly variations of phonon frequency and phonon spectra under high density photoexcitation, providing key knowledge for light-induced structure modification^32^,^33^. Time-resolved spectroscopy has been adopted to study the coherent lattice dynamics in 1D^40^,^41^ and 2D materials^42^,^43^,^44^,^45^,^46^,^47^, providing key information on the electron/exciton-phonon interaction, the interlayer coupling, and nonlinear lattice dynamics.

In this work, we present an ultrafast spectroscopic study on the coherent vibrational dynamics in WTe_2_ crystals. We have observed the oscillation signal in time domain originating from the lowest energy A_1_ optical phonon mode as verified by polarization- and wavelength-dependent measurements. With increasing temperature, the oscillation frequency decreases due to the decay of optical phonons into acoustic phonons. The asymmetric phonon spectral profile and a redshift of phonon frequency have been observed with increasing excitation density, implying that the softening of optical phonons is caused by the lattice anharmonicity induced by intense electronic excitation.

## Results and Discussion

### Coherent vibrational dynamics

**Figure 1:**
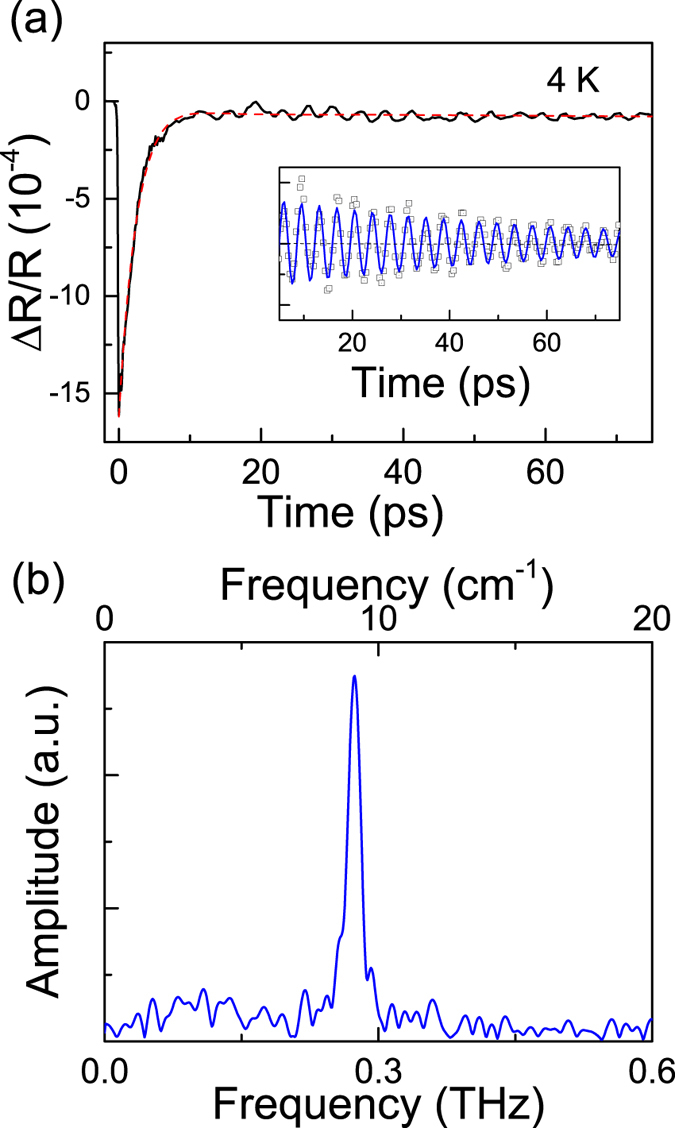
Coherent vibrational dynamics. (**a**) The transient reflectivity signal as a function of time delay in WTe_2_ at T = 4 K. The probe beam at 780 nm is polarized perpendicularly to the pump beam. The pump fluence is 200 μJ/cm^2^. Inset shows the oscillatory component obtained by subtracting off the multi-exponential decay components (the dashed line). (**b**) Fourier transformation of the oscillation component shows the oscillation frequency at 0.27 THz (~9.0 cm^−1^).

**Figure 2:**
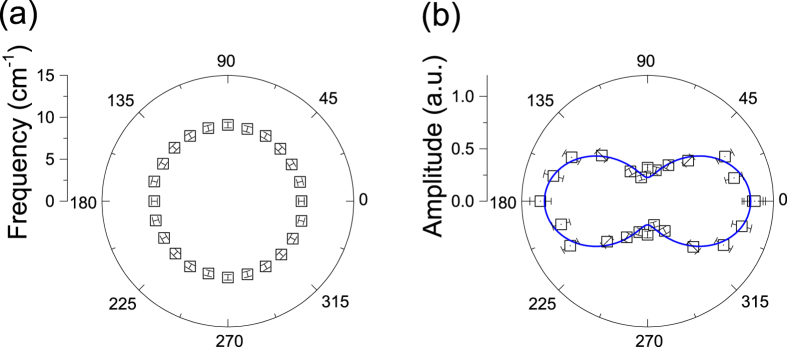
Polarization dependence. Polar plots of the oscillation frequency (**a**) and amplitude (**b**) versus probe polarization angle with respect to the a-axis of WTe_2_ crystals. The pump fluence is 200 μJ/cm^2^.

Figure 1(a) shows the transient reflectivity signal (ΔR/R) as a function of time delay recorded from the WTe_2_ crystal sample at 4 K. The pump and probe beams were set to be cross polarized. Following an abrupt change in reflectivity, the time-dependent signal is manifested as a multi-exponential decay curve entangled with certain periodic oscillations. The exponential decay components are related to the recovery dynamics of photo-excited carriers in WTe_2_^8^. We subtract off the exponential decay parts to analyze the coherent vibrational dynamics in WTe_2_ crystals. The residual oscillations [inset, Fig. 1(a)] can be reproduced by a damped sinusoidal function in the form of^48^,^49^


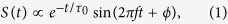


where *f* is the oscillation frequency, *ϕ* is the initial phase, and *τ*_*0*_ is the damping lifetime, respectively. The frequency of oscillation quantified by Fourier transformation (Fig. 1(b)) shows a sharp peak at ~0.27 THz (~9.0 cm^−1^). This energy is close to that of the A_1_ optical phonon with lowest energy as predicted by first-principle calculations^20^,^21^, suggesting the oscillatory component is likely due to the coherent optical phonons. In principle, the lowest energy A_1_ optical mode has a two-fold symmetry in WTe_2_ crystal with *C*_*2v*_ point group^20^,^21^. Polarization-dependent measurements have been performed to check the symmetry of the oscillation signal in time domain. The oscillation behavior is insensitive to the pump polarization but is strongly dependent on the probe polarization (Fig. 2). Figure 2 shows the dependence of oscillation signal on the probe polarization. The frequency of oscillation remains nearly identical (Fig. 2(a)) but the amplitude shows strong polarization dependence with a two-fold symmetry (Fig. 2(b)) which is expected for A1 optical phonon. Regarding this, it is reasonable to assign the observed oscillation as due to the coherent dynamics of the A_1_ optical phonon. The experimental traces show some disparity from the damped sinusoidal function [Supplementary Fig. 2], which is possibly caused by the nonlinear lattice dynamics as we discussed in the following section.

In the concerned regime of phonon energy, coherent acoustic phonons have also been frequently observed as oscillations in time domain. In bulk materials, the light reflection by the propagating strain pulse may interfere with the portion of probe beam reflected from the sample surface. Such a coherent acoustic phonon behavior may also induce oscillations in time domain. To exclude this effect, we have conducted wavelength-dependent measurements on the oscillatory component. For coherent acoustic phonons, the oscillations seen in the transient reflectivity are caused by the temporal evolution of the phase difference that varies in time. For quasi-normal incidence, the frequency of the oscillation is inversely proportional to the probe wavelength^48^,^50^,^51^, i.e.,


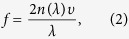


where *n*, *υ*, and *λ* are the refractive index, the propagating velocity of the strain pulse, and the probe wavelength, respectively. Taking the theoretical value of sound velocity^52^, Eq. (2) predicts the frequency is about one-order magnitude lower than the experimental data, suggesting the coherent acoustic phonon is not responsible for the oscillation we observed here. This assignment is also confirmed by the wavelength-dependent experiments (Supplementary Fig. 1). The frequency is nearly independent of the probe wavelength, so that it is safe to exclude the coherent acoustic phonons from being the origin of the oscillation observed here.

### Temperature dependence

**Figure 3:**
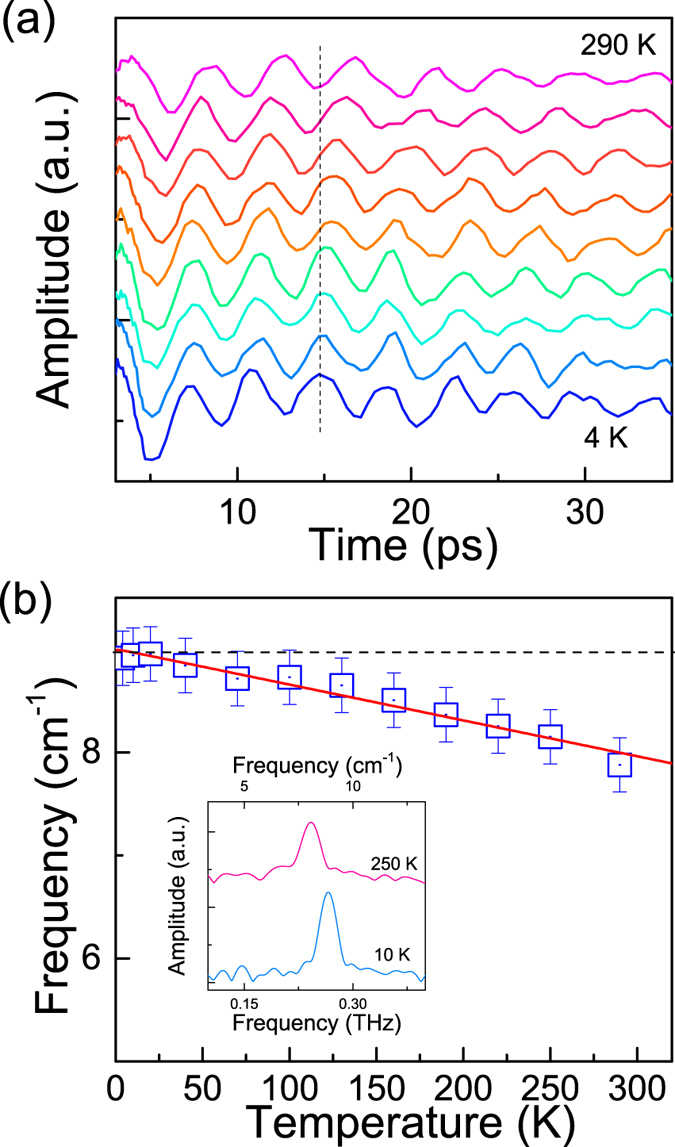
Temperature dependence. (**a**) The oscillatory components probed at different temperatures. The curves are vertically shifted for clarity. (**b**) Temperature-dependent frequency of the oscillation mode. Inset shows the Fourier transformed spectra obtained from time-domain data in (**a**) at 10 and 250 K. The pump fluence is 200 μJ/cm^2^.

The above results of polarization- and wavelength-dependent experiments support the assignment of the oscillations to the coherent behavior of A_1_ optical phonon mode. To gain more information of this mode in WTe_2_ crystals, we carried out the temperature-dependent measurements. Figure 3(a) plots the extracted oscillatory component recorded from 4 to 290 K. With increasing temperature, the oscillation period gradually increases (Fig. 3(a)) while the amplitude slightly drops. The temperature dependence of oscillation frequency is highlighted in Fig. 3(b), which provides a clear evidence for the softening of optical phonon modes as observed in Raman spectroscopy in other transition metal dichalcogenides^53^. For Raman spectroscopic study, the temperature-dependent shift in phonon energy has been frequently modelled by considering the decay of optical phonons into acoustic phonons with the same frequency but opposite momenta^20^,^48^,^54^. In this model, the temperature-dependent phonon energy can be expressed in the form of^20^,^48^,^54^


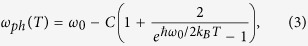


where *C* is a positive constant and *ω*_*0*_ is the bare phonon frequency. For comparison, the data in the study can be well produced by Eq. (3) with *ω*_*0*_ = 9.1 cm^−1^ and *C* = 0.011 cm^−1^.

### Phonon softening

**Figure 4:**
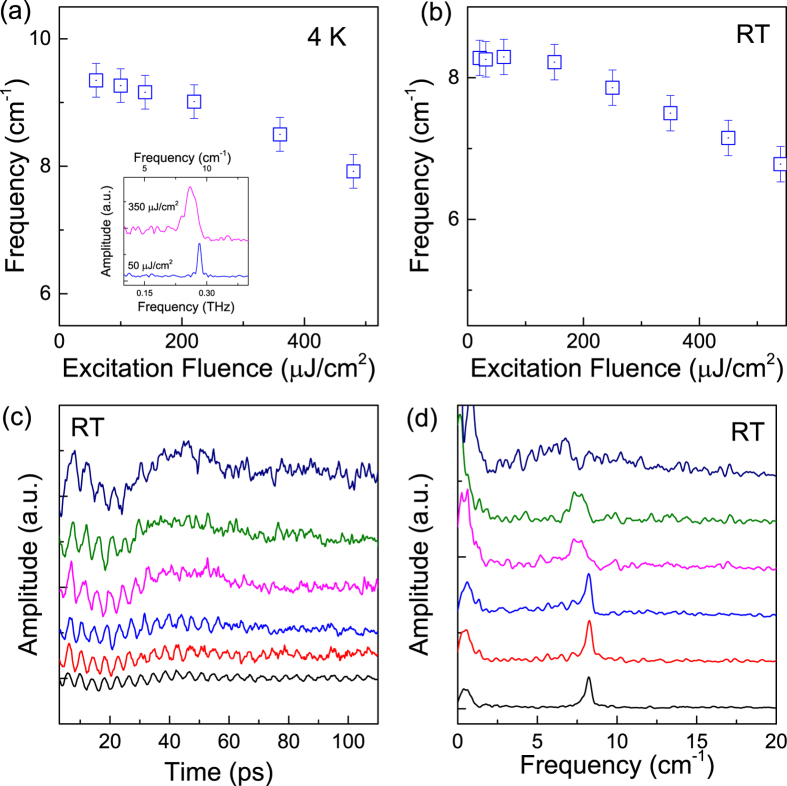
Phonon softening. (**a**) The peak frequency of the A1 mode as a function of the excitation fluence recorded at 4 K. Inset shows the Fourier transformed spectra obtained from time-domain data recorded under relatively low and strong fluence excitations, respectively. (**b**) The peak frequency of the A1 mode as a function of the excitation fluence recorded at room temperature. The oscillation components (**a**) and the Fourier transformed spectra recorded at room temperature with excitation density from 20 to 540 μJ/cm^2^ (from bottom to top), respectively. The curves are vertically shifted for clarity.

In the Raman spectroscopic study on WTe_2_ crystals, the profiles of all optical phonon modes have been reported to be symmetric^20^. The spectra of coherent phonons in frequency domain (Figs 1(b) and 3(b)) show a slight asymmetric peak with a little broader profile at the low-energy side recorded with an excitation of 200 μJ/cm^2^. In previous works on coherent vibrational dynamics on bismuth samples, it has been argued that the asymmetric profile in the frequency domain could be generated by high density excitations^32^. We have carried out measurements under different excitation fluences and observed clear power dependences of the profile and peak frequency of the phonon spectra (Fig. 4). The phonon spectrum recorded under a relatively weak excitation is symmetric at 4 K but the profile under strong excitation shows a significantly asymmetric shape (Inset, Fig. 4a).

In principle, thermal effect caused by laser heating may also involve in phonon softening (Fig. 3) particularly in the low temperature regime where the thermal capacity of WTe_2_ is low (Supplementary Note 1). To minimize the thermal effect, we carried out the experiment at room temperature where the temperature increase is less than 25 K under excitation of 500 μJ/cm^2^ due to relatively large thermal capacity (Supplementary Note 1). Under weak excitation, the oscillatory component is entangled with a slow damped oscillation due to coherent acoustic phonon (Supplementary Fig. 3). The slight asymmetry in the spectral profile of the coherent phonons may be relevant to lattice anharmonicity due to the phonon-phonon interaction (Supplementary Fig. 3).

The pump-probe traces under excitation with different densities recorded at room temperature are shown in Supplementary Fig. 4. With increasing excitation, the frequency of coherent phonons also decreases at room temperature (Fig. 4b). A large softening effect with a 15% variation in phonon frequency of the A_1_ optical mode in WTe_2_ with the excitation fluence up to 500 μJ/cm^2^ (i.e., carrier density of ~3.3 × 10^20^ cm^−3^) (Fig. 4b). In addition, the damping of the oscillation becomes stronger with increasing pump density, manifested with a more asymmetric and broader spectral profile (Fig. 4c–d). The results suggest a high degree of lattice anharmonicity under intense excitation, implying possible involvement of electronic softening effect. From the dynamic point of view, the electronic softening effect is reasonable since the oscillation period is shorter than the lifetime of photo-excited carriers in WTe_2_^8^. The fluence-dependent behavior of oscillatory signal is similar to the amplitude modes observed in some charge-density wave (CDW) systems^55^,^56^. A recent theoretical work suggests CDW may be important in stabilizing the structure of WTe_2_^57^, implying the possible existence of CDW like in other transition metal dichalcogenides^58^,^59^. The oscillatory mode observed here is not a CDW amplitude mode, but the possible mixing-down of phonon mode and CDW to zero wave vector may result in similar behavior^60^, which deserves more in-depth study in future.

With either raising temperature or increasing excitation fluence, the oscillation frequency shows a similar decreasing trend. However, the underlying mechanisms responsible for the frequency shift in the two cases are probably different. To highlight the difference, we compare the fluence dependence and temperature dependence of the oscillation amplitude in Supplementary Fig. 5. With changing temperature, the amplitude of oscillation varies with the signal of differential reflectivity, and the amplitude ratio between the oscillation component and differential reflectivity remains nearly unchanged (Supplementary Fig. 5(d–f)). In contrast, a strong nonlinear dependence of the oscillation amplitude on the fluence has been observed. With changing excitation fluence, the fluence dependence of oscillation amplitude is different from that of differential reflectivity (Supplementary Fig. 5(a,b)), and the ratio between the amplitudes of oscillation component and differential reflectivity gradually decreases (Supplementary Fig. 5c). Such fluence dependence is probably relevant to the structure instability due to nonlinear lattice dynamics induced by strong electronic excitation. The oscillatory component is detectable only when lattice vibrates coherently. Intense excitation may drive a large lattice distortion as evidenced by the frequency shift. In this case, the interaction of the coherent phonons with the unperturbed lattice may cause a strong damping of vibrational coherence, manifesting as the amplitude saturation and strong damping of the oscillatory components.

## Conclusions

In summary, we have studied the coherent vibrational dynamics in WTe_2_ crystals by employing ultrafast pump-probe spectroscopy. The polarization- and wavelength-dependent measurements support that the oscillation component of the signal in time domain originates from the coherent dynamics of the lowest energy A_1_ optical phonon mode in WTe_2_. The oscillation frequency decreases with increasing temperature which can be well explained by the standard model of anharmonic decay of optical phonons. Moreover, a large phonon softening is identified in WTe_2_ where the phonon energy drops significantly with increasing pump power due to the lattice anharmonicity induced by intense excitation. The nonlinear lattice dynamics and the large electronic effect on the coherent phonons observed in WTe_2_ are meaningful for better understanding fundamental physics in the emergent systems including the lattice dynamics and the structure stability in low dimensional systems^42^,^43^,^44^,^61^,^62^. Since the A_1g_ mode is also infrared active, we speculate that it may be possible to control the GMR effect through vibrational excitation with ultrashort pulse^63^,^64^.

## Methods

### Sample preparation

The single crystals of WTe_2_ were grown by a chemical vapor transport method^1^. The WTe_2_ polycrystalline was first synthesized by tungsten and tellurium powders at 750 °C. Then about 2 g WTe_2_ powder was filled in the quartz tube with a 3 mg/ml Br_2_ as a transporting agent. The quartz tube was evacuated and sealed, then stayed for two weeks in a two-zone furnace with a temperature gradient between 750 to 650 °C to grow large single crystals. The crystalline axes were identified by X-ray diffraction and optical microscopy. The crystals were cleaved in air to obtain flat surfaces and then transferred into a Helium-free cryostat system (OptistatAC-V12, Oxford Instruments).

### Optical characterizations

The transient reflectivity measurements were performed with a Ti:sapphire regenerative amplifier (Libra, Coherent Inc.). The fundamental output at 800 nm was used as the pump beam while an optical parametric amplifier system (Opera Sola, Coherent Inc.) pumped by the regenerative amplifier was employed as the probe beam for wavelength-dependent measurements. The spot sizes for pump and probe beams at the sample were ~1 mm and 0.5 mm, respectively. In our experiments, the probe intensity was about one order of magnitude weaker than that of the pump pulse. A half-wavelength waveplate was used for the polarization-dependent experiments.

## Additional Information

**How to cite this article**: He, B. *et al*. Coherent optical phonon oscillation and possible electronic softening in WTe_2_ crystals. *Sci. Rep.* **6**, 30487; doi: 10.1038/srep30487 (2016).

## Supplementary Material

Supplementary Information
