# Supplementary material for: Coherent optical phonon oscillation and possible electronic softening in WTe2 crystals
Source: Sci Rep. 2016 Jul 26;6:30487. doi: 10.1038/srep30487 (PMC4960623; doi:10.1038/srep30487)
Supplement: Supplementary Information [file srep30487-s1.pdf]

Coherent optical phonon oscillation and possible electronic softening in WTe_2 crystals

Bin He,[1] Chunfeng Zhang,[1, 2, a] Weida Zhu,[1] Yufeng Li,[1] Shenghua Liu,[1] Xiyu Zhu,[1] Xuewei Wu,[1] Xiaoyong Wang,[1] Hai-hu Wen,[1] and Min Xiao[1,2, 3,b]

[1]National Laboratory of Solid State Microstructures, School of Physics, Nanjing University, Nanjing 210093, China
[2]Synergetic Innovation Center in Quantum Information and Quantum Physics, University of Science and Technology of China, Hefei, Anhui 230026, China
[3]Department of Physics, University of Arkansas, Fayetteville, Arkansas 72701, United States

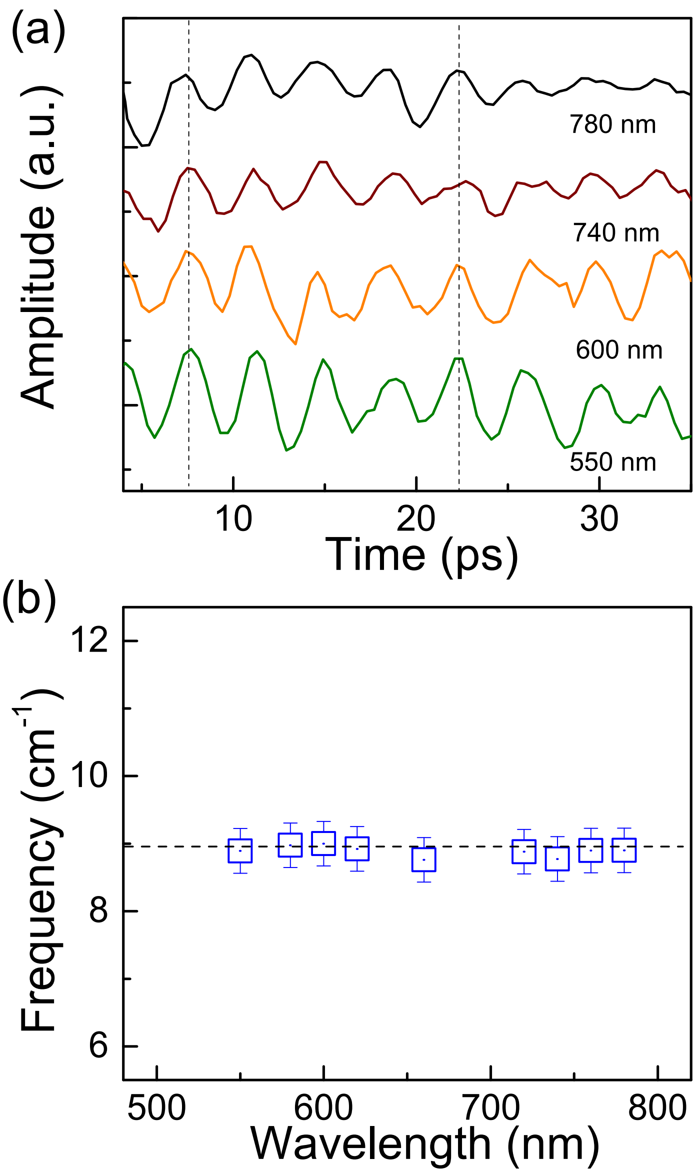

Supplementary Figure 1. Probe wavelength dependence. (a) The oscillatory components probed at different wavelengths. The curves are vertically shifted for clarity. (b) The oscillation frequency is plotted as a function of the probe wavelength. The pump fluence is 200 μJ/cm^2.

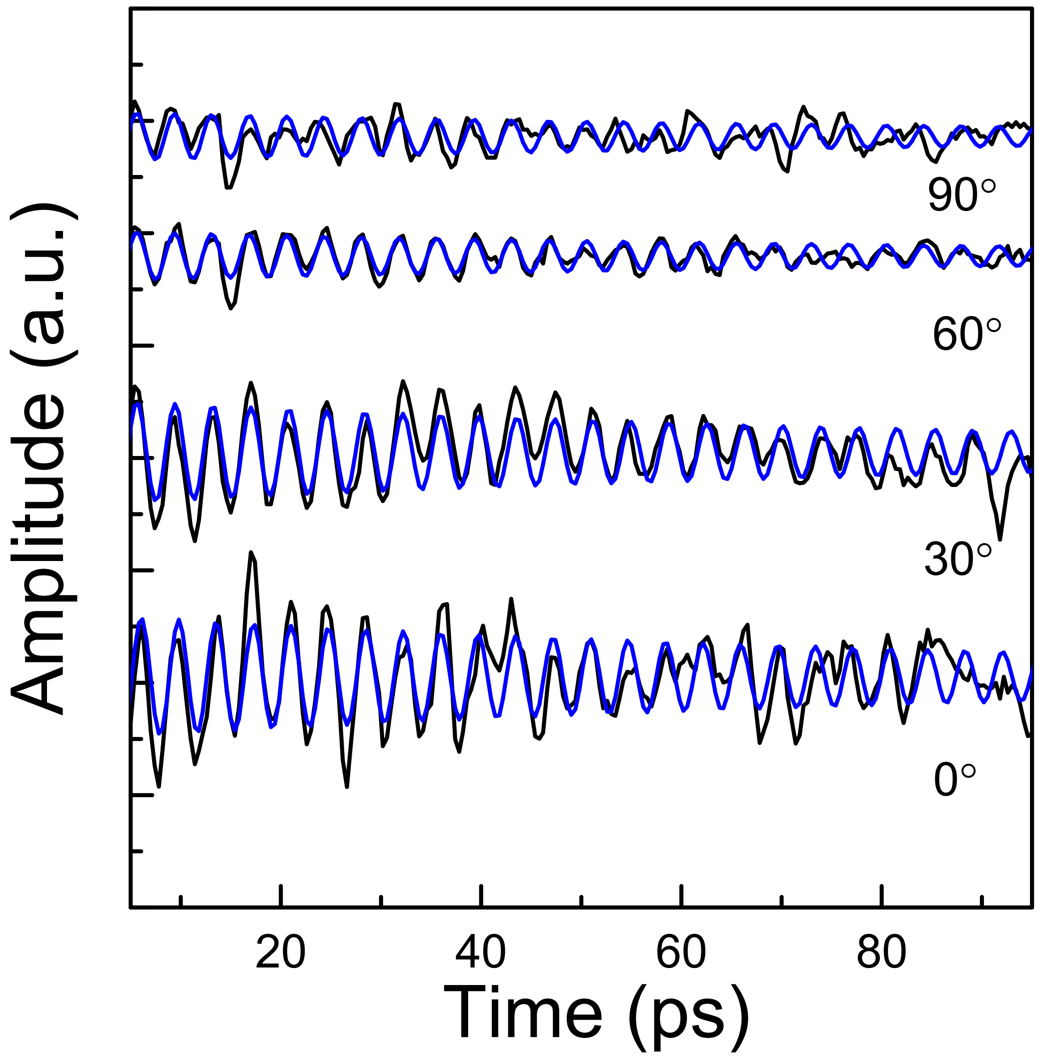

Supplementary Figure 2. Polarization dependence. The oscillatory component probed at different polarization angles with respect to the a-axis of WTe_2 crystals. The black and blue lines are experimental data and curves fitting to the damped oscillation function.

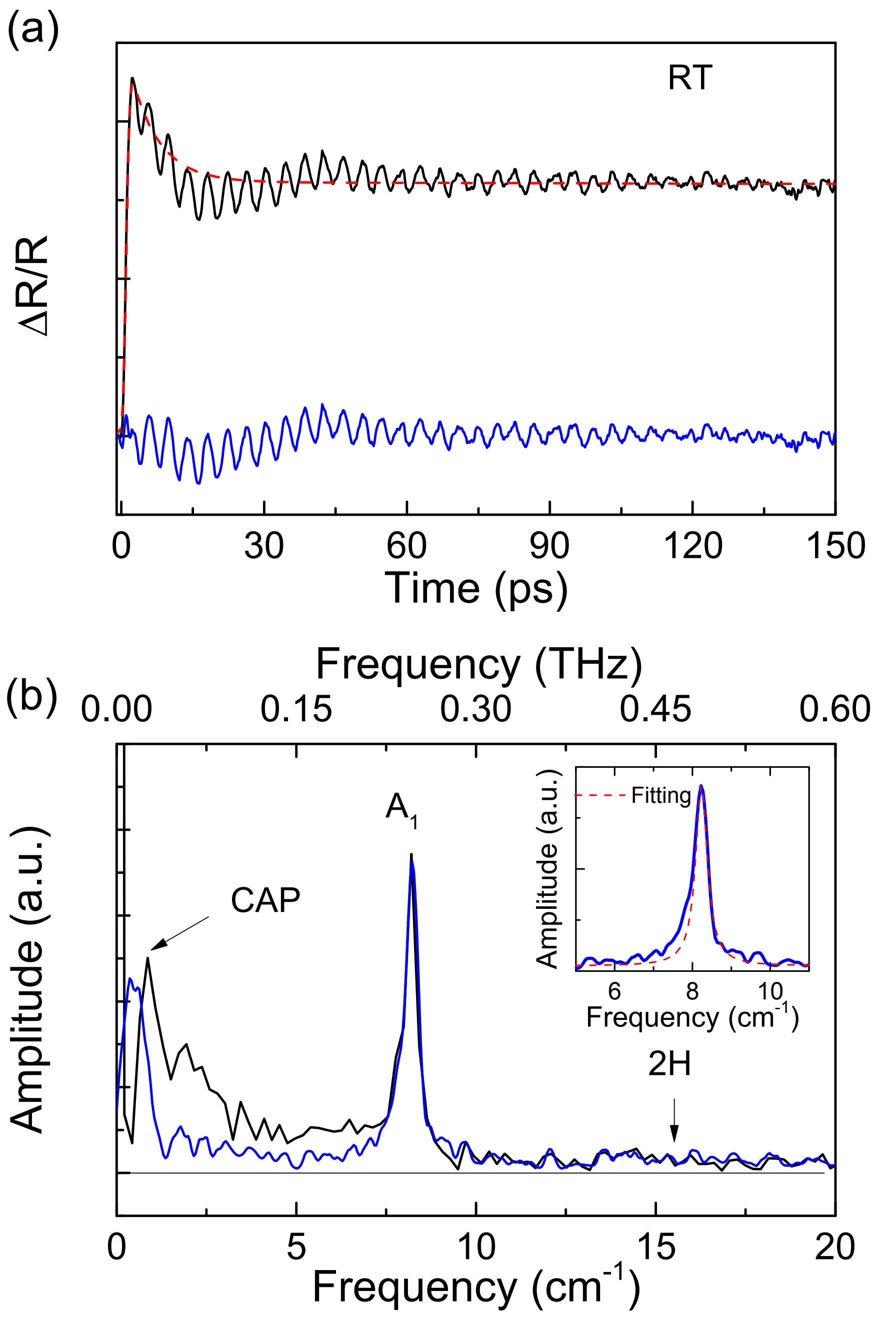

Supplementary Figure 3. (a) The pump-probe trace recorded with excitation fluence at 60 μJ/cm^2 (black). The dashed line is the biexponential fitting curve. The blue is the oscillatory component obtained by subtracting the biexponential decay from original curve. (b) The Fourier transformed spectra from time-domain data of the original curve (black) and the subtracted curve (blue). Inset shows the spectrum of A1 optical mode in comparison with the Lorentz peak profile. In addition to the A1 optical mode, the low frequency oscillation mode is likely to be relevant to the coherent acoustic phonons (CAP). No significant peak is detected at the 2nd harmonics of A_1 mode, implies the anhamonicity is induced by coupling between A1 mode and acoustic phonons.

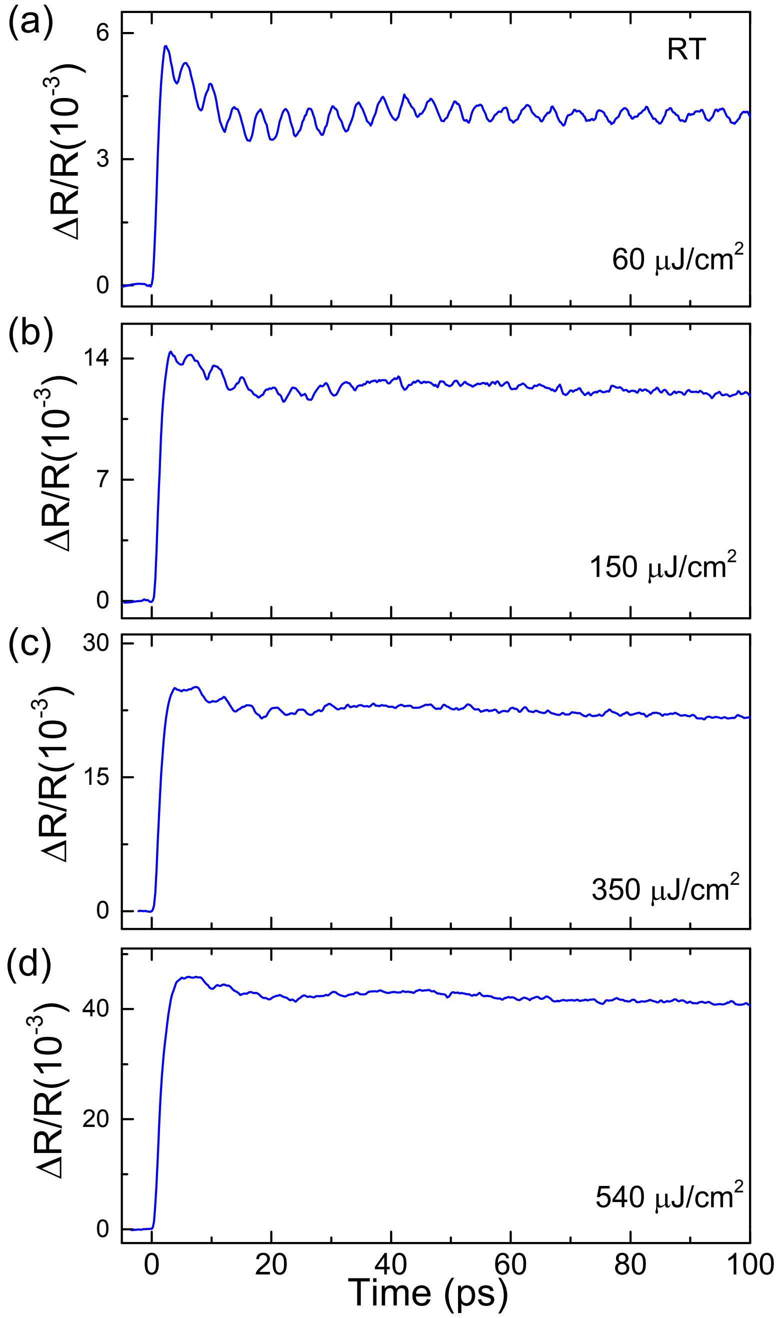

Supplementary Figure 4. Fluence-dependent coherent vibrational dynamics. The transient reflectivity signal is plotted as a function of time delay in WTe_2 recorded with different excitation fluences at room temperature. The visibility of oscillatory component decreases with increasing excitation fluence. The data were recorded with perpendicular polarizations of pump and probe beams.

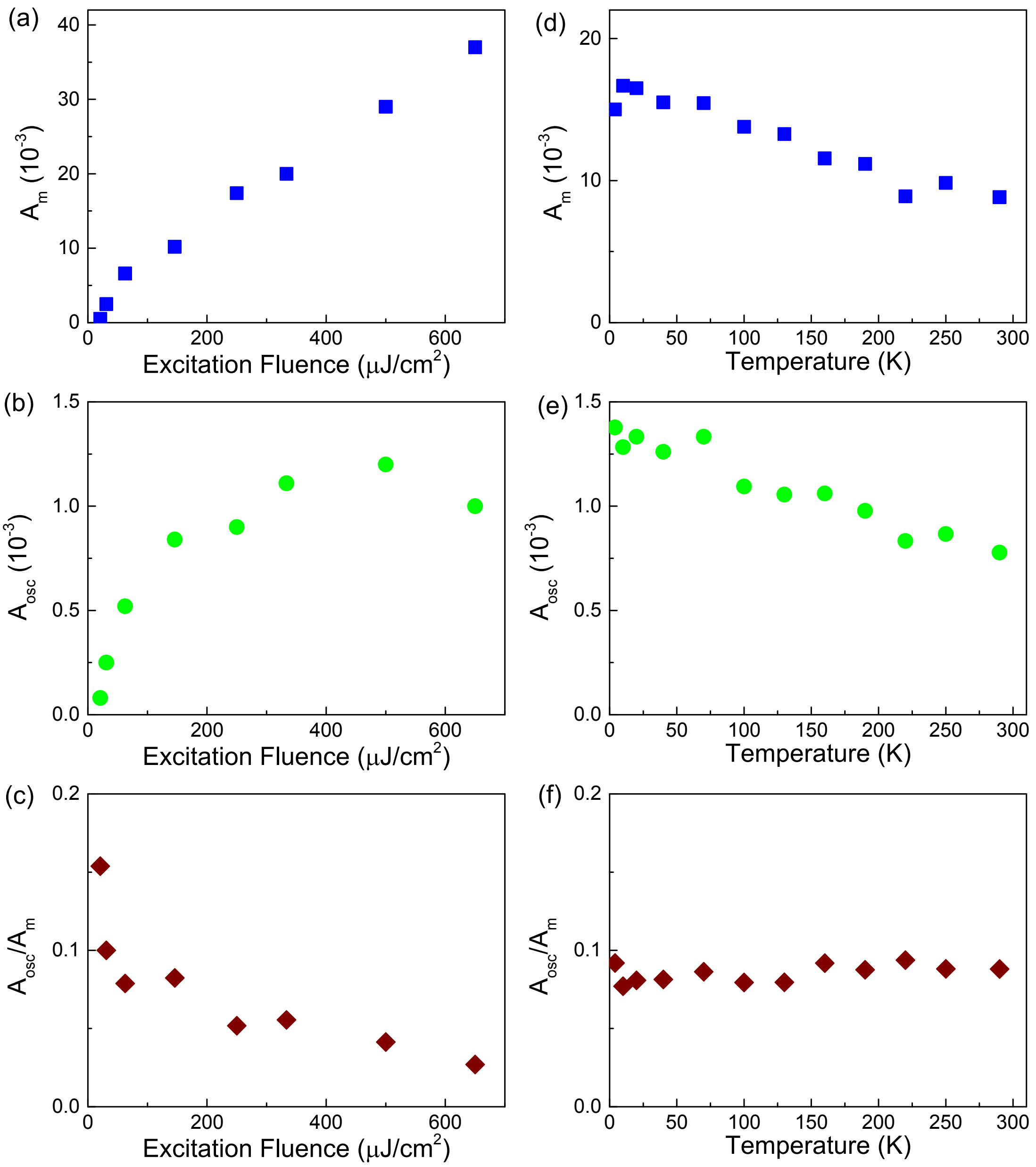

Supplementary Figure 5. Fluence dependence and temperature dependence. (a-c) The amplitudes of differential reflectivity (A_m) and oscillatory components (A_{osc}), and their ratio (A_{osc}/ A_m) are plotted versus excitation fluence at room temperature; (d-f) $A_{m,}$ A_{osc} and A_{osc}/ A_m are plotted versus temperature with excitation fluence of ~ 200 200 μJ/cm^2. The data were recorded at room temperature.

Supplementary Note 1.

We evaluated the carrier density and possible laser heating effect by considering the light absorption of WTe_2 at 800 nm. From the literature value of optical constants,[1] the absorption coefficient is estimated to be $\alpha \sim 1.6 \times 10^5$ cm^{-1}. The excitation penetration depth is then approximated to be $\boldsymbol{L} \sim 60$ nm. The reflectivity at the surface of WTe_2 is R ~ 42% at 800 nm. With excitation fluence of $\boldsymbol{I} = 500$ μJ/cm^2, the carrier density can be calculated to be ~ 3.3×10^{20} cm^{-3}.

We neglect the thermal diffusion to roughly estimate the transient temperature jump induced by laser heating. For the excitation at a low frequency (1 KHz), the pulse-to-pulse thermal accumulation can be neglected. We assume that all the energy deposited on the sample was transferred to heat. In this case, the temperature jump (ΔT) at temperature (T_0) can be calculated as

$$\frac{SL\rho}{M}\int_{T_0}^{T_0+\Delta T} C(T)\,\mathrm{d}T = (1-R)IS\,, \quad [1]$$

where *C(T)*, *S*, *ρ* and *M* are the temperature-dependent thermal capacity, the excitation area, the mass density (9.43 g cm^{-3}) and the molar mass (439 g mol^{-1}) of WTe_2. With excitation fluence of 500 μJ/cm^2, we estimated the values of ΔT to be ~ 80 K and ~ 25 K with T_0 at 4 K and 300 K, respectively, by adopting the literature values of thermal capacity of WTe_2.[2] Even at 4 K, the temperature jump induced phonon softening (Figure 3) is much lower than that observed experimentally (Figure 4), suggesting a key role played by electronic effect.

Supplementary References

1 Homes, C. C., Ali, M. N. & Cava, R. J. Optical properties of the perfectly compensated semimetal WTe2. *Phys. Rev. B* **92**, 161109 (2015).

2 Callanan, J. E., Hope, G. A., Weir, R. D. & Westrum, E. F. Thermal properties of Tungsten Dietelluride (WTe_2). 1. The preparation and low-temperature heat capacity at temperatures from 6 K to 326 K. *J. Chem. Thermodyn.* **24**, 627-638 (1992).
